# Codon usage: Nature's roadmap to expression and folding of proteins

**DOI:** 10.1002/biot.201000332

**Published:** 2011-06

**Authors:** Evelina Angov

**Affiliations:** Division of Malaria Vaccine Development, Walter Reed Army Institute of ResearchSilver Spring, MD, USA

**Keywords:** Codon usage, Protein folding, Translation

## Abstract

Biomedical and biotechnological research relies on processes leading to the successful expression and production of key biological products. High-quality proteins are required for many purposes, including protein structural and functional studies. Protein expression is the culmination of multistep processes involving regulation at the level of transcription, mRNA turnover, protein translation, and post-translational modifications leading to the formation of a stable product. Although significant strides have been achieved over the past decade, advances toward integrating genomic and proteomic information are essential, and until such time, many target genes and their products may not be fully realized. Thus, the focus of this review is to provide some experimental support and a brief overview of how codon usage bias has evolved relative to regulating gene expression levels.

## 1 Introduction

Biomedical and biotechnological research relies on processes leading to the successful expression and production of key biological products. High-quality proteins are required for many purposes, including protein structural and functional studies. Protein expression is the culmination of multistep processes involving regulation at the level of transcription, mRNA turnover, protein translation, and post-translational modifications leading to the formation of a stable product. Although significant strides have been achieved over the past decade, advances toward integrating genomic and proteomic information are essential, and until such time, many target genes and their synthetic potential may not be fully realized. Thus, the focus of this review is to provide some experimental support and a brief overview of how codon usage bias has evolved relative to regulating gene expression levels.

Due to their apparent “silent” nature, synonymous codon substitutions have long been thought to be inconsequential. In recent years, this long-held dogma has been refuted by evidence that even a single synonymous codon substitution can have significant impact on gene expression levels, protein folding, and protein cellular function [1–4]. It is certainly conceivable that, by design, nature has provided the basic instructions to direct efficient protein synthesis and folding through the information encoded at the genetic code level. For most se-quenced genomes, synonymous codons are not used at equal frequencies. Sixty-one codons specify the twenty amino acids found commonly in protein sequences; most of these are specified by more than one synonymous codon, with the exception of methionine and tryptophan. The redundancy in the genetic code may have evolved as a way to preserve structural information of proteins within the nucleotide content [5]. In unicellular organisms, high-frequency-usage codons correlate with abundant cognate isoacceptor tRNA molecules and have evolved to optimize translational efficiency [6–10]. In bacteria, the co-evolution of tRNA isoacceptor abundance and codon bias is most evident for proteins from highly expressed genes involved in essential cellular functions, such as protein synthesis and cell energetics, and are likely to have co-developed as a result of a positive selective force to achieve faster translation rates and translational accuracy [7, 8, 11–13]. A similar relationship between codon usage bias and intracellular tRNA abundance levels on translation efficiency is also observed for some eukaryotes, *C. elegans* and *D. melanogaster* [14, 15]. Although somewhat controversial, evidence for the ubiquity of codon bias functionality in translational control was recently extended to the tissue-specific level in eukaryotes [16–19].

Codon bias has been extensively observed and varies widely within genomic DNA sequences of different organisms [6, 7, 20, 21]. Even with relatively limited genomic sequence data, early studies in prokaryotes and yeast established the fundamental existence of codon bias in encoded DNA [21–24]. From these earlier studies, a positive correlation between codon usage, gene expression level, and growth efficiency of prokaryotic cells [25] was established and used to generate codon adaptation indices (CAI) [26]. CAI define a relative “adaptiveness” to codons and are widely used to predict expression levels from genes and to approximate the success of heterologous gene expression. A caveat and limitation is that the predictive value of CAI is directly dependent on the genes used to establish the “reference set” and may have relatively limited value for predicting expression from genes not reflected by the codon bias found in the reference set. Recognizing these limitations, investigators have developed more “universal” CAI, which measure codon bias based on reference sets that are not necessarily derived from preferred, “highly” expressed genes, but from all known coding sequences for a specific organism [27]. Notwithstanding these limitations, CAI do not necessarily reflect all possible factors that influence gene expression levels per se, for example, the efficiency of ribosome binding and translation initiation [28]. Similar selective forces on codon co-adaptation to tRNA pools have also been seen for some eukaryotes, namely, yeast [21], Drosophila [29, 30], *Caenorhabditic elegans* [31], *Arabidopsis thaliana* [32], and *Xenopus laevis* [33]. Unlike prokaryotes and some eukaryotic organisms, the evolutionary pressure on codon bias in the human genome cannot be solely explained by forces on selection for translational efficiency [16, 34], but are also influenced by the extensive guanidine:cytosine (GC) content found in isochore structures on chromosomes (GC-rich DNA <100 kb) [35] and by the effects of mRNA secondary-structure stability [36].

The recent volume of completed nucleotide sequence and protein structural data for many species has allowed for comprehensive analyses into the relationship between genomic GC content, codon usage bias, and gene expression levels. Knight et al. examined the codon usage pattern for a large set of species (311 prokaryotes, 28 archaea and 257 eukaryotes) using a relatively simple quantitative mutational model and predicted that the GC mutational bias on genomes rather than codon usage bias on GC content was the driving force for both codon and amino acid usage in prokaryotes and eukaryotes [37]. These conclusions were strengthened by the replication of the observations across all three cellular life domains, archaea, bacteria, and eukaryota, suggesting highly conserved mechanisms for mutational and selection equilibrium at the genome level. These findings were corroborated in a more recent study using a stochastic continuous Markov chain model for GC-biased synonymous point substitutions across hundreds of bacterial, plant, and human genes. The model established that GC mutational bias is indeed a dominant factor determining codon bias [38]. However, other factors could account for the codon bias of an organism not considered within the model, for instance, transcriptional efficiency in bacteria and the GC skew in mammals (GC isochores).

## 2 Role of mRNA structure on gene expression

It is widely held that mRNA secondary structure influences translational efficiency. In bacteria, formation of strong hairpin loops centered at the Shine-Dalgarno (SD) ribosome binding site (RBS) and the initiation codon (AUG) can significantly reduce expression levels [39]. With regards to heterologous protein expression, synonymous codon substitution at the 5′-end of mRNA can impact mRNA structure and stability and thus the relative kinetics of translation at both the level of translation initiation [40] and elongation [41]. As such, there are at least two influences at the nucleotide level that govern regulation of expression from the 5′-end of open reading frames (ORFs): the GC content and formation of mRNA secondary structure (discussed here), and codon usage bias and efficiency of translation initiation (discussed later in the context of slowing translation). Gu et al. reported that mRNA secondary-structure stability correlated with both GC content and codon usage [42]. In their study of 340 genomes from bacteria, archaea, fungi, plants, insects, fishes, birds, and mammals, they revealed that, with the exception of birds and mammals, the 5′-end translation initiation sites had reduced mRNA secondary structure. The universality of their observations suggested that reduced mRNA stability at 5′-ends of ORFs may have evolved as a result of selective pressure toward efficient translation initiation. These observations were further expanded by Allert et al., who analyzed the nucleotide composition of 816 fully sequenced bacterial genomes [43]. They found that nucleotide composition at both the 5′- and 3′-ends of an ORF played an important role in gene expression levels. Their analysis revealed a bias toward higher adenine:thymidine (AU) content in the first and last 35 bases of an ORF relative to the central coding region of the ORF. Not surprisingly, the highest expression levels were observed when all three parameters (AU content, mRNA secondary-structure content, and CAI) were considered at the same time. By using a systematic approach, the impact of random, synonymous codon substitutions on mRNA secondary-structure stability was experimentally derived using 154 gene variants expressing green fluorescent protein (GFP) in *E. coli* [44]. These investigators concluded that synonymous codon substitutions that reduced mRNA structure stability, particularly in the first forty nucleotides of the transcript, were significantly correlated with GFP protein abundance. They attributed the majority of the effect on GFP expression to the local nucleotide content and not to codon usage bias or CAI. Supek and Smuc [45] recently refuted Kudla et al. [44] using nonlinear regression analysis on the same data and argued that the effects of CAI and codon bias were masked by the inherent strong mRNA structure found in GFP. They argued that codon usage may have a significant role in gene expression levels, particularly in cases in which 5′-mRNA is weakly associated with secondary structure [45].

## 3 Role of rare codons in gene translation

Mounting evidence exists that low-frequency-usage codons within a coding sequence can provide the genetic instruction that regulates the rate of protein synthesis to allow for some secondary and tertiary structure formation by the nascent polypeptide [2, 46]. Computational analysis of the available *E. coli* genome and protein structure databases identified that high-frequency-usage codons are mainly associated with structural elements, such as alpha helices, whereas clusters of lower frequency usage codons are more likely to be associated with beta-strands, random coils, and structural domain boundaries [47]. These findings confirmed earlier speculations that the positioning and clustering of codons with different usage frequencies was non-random and played a role in gene expression [6, 8, 48].

Some experimental evidence for the role of synonymous codon substitutions, particularly in slow translating regions on mRNA, and their impact on protein structure or function are briefly summarized. Systematic single-codon substitutions of five low-frequency codons in a linker region of the *Echinococcus granulosus* fatty acid-binding protein 1 (EgFABP1) gene to synonymous higher frequency usage codons significantly impacted protein solubility [49]. These results suggest that codon substitution in a slower translation region alters the kinetics of translation and in vivo folding. In another example, synonymous substitution to eliminate rare codons in chloramphenicol acetyltransferase yielded significantly reduced specific enzyme activity [50], also suggesting a dramatic effect on folding. In the framework of heterologous gene expression, deleteriously placed translational pause sites have also been shown to lead to translational frame-shifting [51, 52] and to protein misfolding [53]. A single silent mutation in the human MDR1 gene caused the P-glycoprotein to have an altered folding pathway. Other studies also point to low-frequency-usage codons in “pausing” translation to allow local protein-structure formation [18, 54, 55].

Alternatively, targeted substitution to introduce low-frequency codons at the 5′-end of a coding sequence enhanced heterologous expression levels for streptokinase from the *src* gene of *Streptococcus equisimilis* in *E. coli* [56] .Arguably similar to the recent findings by Tuller et al., inclusion of rare codons at key positions allowed for stabilization of the ribosomal initiation complex, or concomitantly, reduced mRNA secondary structure, thereby yielding higher levels of recombinant protein [41]. They reported that the positioning of low-frequency codons to the 5′-end of an ORF is a universally conserved phenomenon and is a putative mechanism for regulating gene expression. A pattern emerges that low-frequency codon bias, particularly at the 5′-end of the ORF, has evolved as a means to regulate the efficiency of translation initiation. In this model, the first 30–50 codons act as the “ramp” for slowing translation initiation to allow for efficient ribosome binding on mRNA and to avoid ribosome bottlenecking. Therefore, selection for “poorly” adapted codons at the 5′-end on mRNA is a mechanism to reduce deleterious ribosomal traffic jams on the messenger and control the rate of peptide elongation [57–59]. Interestingly, the second codon position immediately following the initiation codon, AUG, is more likely to be a high-frequency-usage codon, and thereby, would be translated more quickly. This codon organization would ensure efficient translation initiation, release, and recycling of the initiator tRNA for the next round of initiation. And finally, in the case of bacterial exported proteins, rare codon clusters at the 5′-end of the ORF may allow time for the nascent translocating peptide–ribosome complex to reach target cellular membranes [60]. In prokaryotes, these rare codon clusters have adapted to act in a similar fashion as the signal recognition particles (SRP) in eu-karyotes.

**Figure 1:**
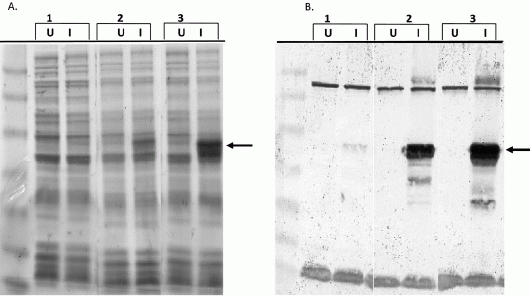
Codon harmonization improves expression level of *P. falciparum* malaria protein, MSP1_42_ in *E. coli*. (**A**) Coomassie Blue-stained gel on total cell lysates, uninduced (U) and induced (I) cells (3 h with 0.1 mM isopropyl β-d-1-thiogalactopyranoside (IPTG)), expressing *P. falciparum* MSP1_42_ from various constructs. (**B**) Western blot on total cell lysates (same as above) probed with rabbit polyclonal anti-MSP1_42_ antibodies for U and I cells (3 h with 0.1 mM IPTG), expressing *P. falciparum* MSP1_42_ from various constructs. Lane 1: MSP1_42_ with a single, synonymous codon substitution; lane 2: MSP1_42_ codon-harmonized at the 5′-end, first thirty codons; and lane 3: MSP1_42_ full gene sequence codon-harmonized. Arrow points to the expressed protein band.

**Figure 2:**
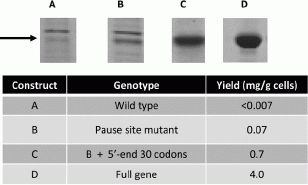
Quantitation of expression levels of *P. falciparum* MSP1_42_ following partial purification. Cells expressing various *P. falciparum* MSP1_42_ constructs were purified by nickel-affinity chromatography. Construct A represents the yield from the native gene sequence of *P. falciparum* MSP1_42_. Construct B represents the yield from the single, synonymous codon substitution (pause-site mutant). Construct C represents the yield from the 5′-end first thirty harmonized codons. Construct D represents the yield from the full gene codon-harmonized sequence. Arrow points to the partially purified band.

From a practical perspective, we applied the codon harmonization approach iteratively to optimize expression levels in *E. coli* for the malaria *P. falciparum*, Merozoite Surface Protein 1, (MSP1_42_). In the first instance, we targeted a putative translational pause site that was predicted to be in disharmony when expressed in *E. coli* [61]. Substitution to harmonize the codon frequency toward that of the native sequence codon resulted in an approximately ten-fold improvement in soluble-protein expression (Fig. 1). Partial purification by nickel-affinity chromatography allowed for a more quantitative comparison of expression levels relative to the native sequence, that is, to compare native and single-pause-site mutant (Fig. 2A and B). Interestingly, in the second instance, when we codon-harmonized the first thirty codons at the 5′-end of the ORF, we saw another dramatic improvement in expression levels (Fig. 1, lane 2, and Fig. 2C). In the final instance, codon harmonization of the full gene sequence of MSP1_42_ yielded the highest level of soluble protein compared with both the single-pause-site mutant and the 5′-end harmonized variants (Fig. 1, lane 3, and Fig. 2D) [62, 63]. These results demonstrate that incremental improvements in expression levels can be achieved, however, at least for the case of this malaria protein, maximal improvements were only achieved when the full gene sequence was recoded for heterologous expression, yielding approximately 1000-fold higher protein yields than the native gene sequence. An excellent review on the role of sequence codon bias and gene expression was recently published by Plotkin and Kudla [4].

## 4 Practical considerations for heterologous expression

Genetic redundancy at the codon level purportedly increases the organisms' resistance to mutations, however, even a single nucleotide change, leading to a synonymous codon substitution, can impact protein expression, protein structure, and/or function [3, 10, 49, 64–70]. From a practical standpoint, the common causes for failures in heterologous gene expression are primarily related to the disparities in codon bias, mRNA secondary structure and stability, gene product toxicity, and product solubility [71, 72]. Currently, it is accepted that non-optimal codon content can limit expression of heterologous proteins due to limiting available cognate tRNAs in the expression host. Introduction of rare codons that are incongruent with the native gene sequence during heterologous expression can lead to reduced translation rates and overall expression levels [73, 74] .The disparities in codon usage can cause significant stress on host-cell metabolic and translational processes. Various strategies have been used to minimize the bias in codon usage for heterologous expression. In bacteria and simple eukaryotes, the observation that highly expressed genes have strong codon bias toward “preferred” codons led to the development of algorithms for “codon optimization” that substitute codons in a target sequence toward preferred high-frequency codons from the expression host. The premise for this approach is that by introducing the most abundant codons throughout the length of the sequence the resulting protein would be expressed at high levels, primarily because cognate isoacceptor tRNA molecules are not rate limiting [71, 72]. This approach has been successful for the heterologous production of some proteins [75], however, in some cases, the high levels of protein expressed have led to the formation of insoluble products sequestered in inclusion bodies [76]. An alternative approach has been to adjust the intracellular tRNA isoacceptor concentrations directly by co-expressing copies of rare tRNA molecules [71, 77–80]. This approach resolves some, but not necessarily all, codon bias issues. A third approach to recode a target gene sequence is to “match” the codon usage bias inherent in the native host more closely when expressed in the heterologous host and is referred to as “codon harmonization” [62]. In this approach, two features are primarily considered: first, that the expression host synonymous codon usage should more closely match that of the native gene host codon usage, and second, that putative nonstructural segments between local alpha-helical content are coded to translate more slowly. Predicting non-structural segments on proteins without structures obtained by crystallography or NMR spectroscopy is highly empirical and is based on the earlier report by Thanaraj and Argos, which identified ten out of twenty amino acids with bulky hydrophobic side chains or side chains that can hydrogen bond to the peptide backbone as being more likely to be found in nonstructural segments for *E. coli* proteins [81]. Slowing ribosomal translation through these regions may allow co-translational folding by allowing nascent flanking structural elements to gain some structure prior to synthesis of the next element. The codon harmonization approach was successfully applied to express several *P. falciparum* malaria target antigens in *E. coli* [62, 82, 83]. A list of recent, although certainly not exhaustive, review articles focusing on heterologous expression of foreign proteins from various host systems is provided [72, 84–92].

**Figure 3:**
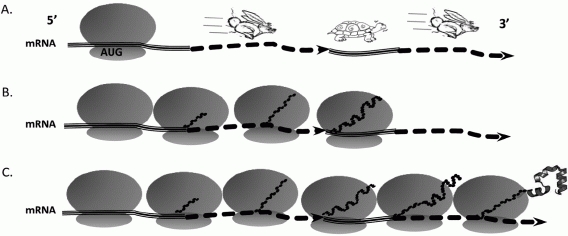
Schematic model of co-translational folding on mRNA by ribosomes. (**A**) Ribosomal complex centered on the translation initiation site, AUG (initiation codon). (**B**) Nascent polypeptide synthesis within the protective environment of the ribosomal tunnel. (**C**) Putative translational pause sites in conjunction with co-translational folding occur within the ribosomal tunnel. Differences in codon usage frequency are shown as thick dashed lines with arrowheads for areas representing high-frequency-usage codons, and therefore, translating rapidly (hare) and regions that are double lined represent segments of lower frequency usage codons (i.e., putative pause sites; tortoise) where translation proceeds more slowly to allow nascent polypeptide folding.

Nascent polypeptide synthesis is complex and is influenced by many factors, such as the rate of tRNA binding, the kinetics of translation and protein folding, the environment within the ribosomal tunnel, and the interaction with chaperones [46, 48, 93, 94]. Efficient protein synthesis and peptide folding occurs co-translationally within the protective environment and is best described for the prokaryotic ribosomal tunnel [95, 96]. Direct interaction of the nascent polypeptide with the tunnel can initiate protein folding by briefly stalling or arresting translation, probably by charge-specific interactions between charged amino acids and the tunnel [97, 98]. In contrast to these direct interactions, the rate of protein translation is also influenced by the local mRNA structure and the presence of slowly translated codons. A crude model representing the kinetics of translation and nascent protein synthesis is shown in Fig. 3. The model depicts (Fig. 3A) a single ribosome binding at the translation initiation complex centered at the AUG codon; at the 5′-end of the mRNA, the double-lined region depicts the “ramp” or slowly translated region; high-frequency codons are translated quickly within the protective ribosomal tunnel (Fig. 3B; tunnel is not shown); and as the translocating ribosome reaches an mRNA segment encoded by low-frequency-usage codons, the rate of translation slows, and allows for the preceding nascent peptide to gain some helical structure within the tunnel (Fig. 3C).

Over the past decade, several codon adaptation algorithms have been developed and are available through public website access (Table 1). Needless to say the success or failure of applying any approach for optimal heterologous protein expression is likely to be sequence dependent and is not necessarily theoretically predictable. Achieving a level of understanding for how genomic codon usage bias has evolved to regulate gene expression in organisms will greatly facilitate the development of synthetic DNA design parameters for optimal heterologous protein production.

**Table 1:** Codon usage analysis and optimization tools

Algorithm	Description	Citation
O_RF_O_PT_	Tunes regional nucleotide composition, codon choice, mRNA secondary structure	[43]
Gene Composer	Gene and protein engineering using PCR-based gene assembly and PIPE cloning.	[100]
Codon Harmonization	Adjusts codon usage by predicting translational pauses and matching codon usage on native gene hosts in heterologous hosts	[62, 63]
GASCO	Codon optimization based on host genome codon bias with the identification of desirable/undesirable motifs http://miracle.igib.res.in/gasco/	[101]
QPSO	Quantum-behaved particle swarm optimization	[102]
OPTIMIZER	Codons computed based on highly expressed prokaryotic genes, based on CAI http://genomes.urv.es/OPTIMIZER	[103]
Gene Designer (DNA 2.0 Inc.)	Synthetic biology workbench using advanced optimization algorithms and an intuitive drag-and-drop graphic interface	[104]
Synthetic Gene Designer	Enhanced functionality enabling users to work with nonstandard genetic codes, with user-defined patterns of codon usage, and an expanded range of methods for codon optimization	[105]
JCat	Codon adaptation with the avoidance of cleavage sites http://ww.prodoric.de/JCat	[106]
GeMS	Gene design functions, including restriction site prediction, codon optimization for expression, stem-loop determination, and oligonucleotide design	[107]
UpGene	SIV/HIV coding sequence adaptation for eukaryotic expression http://www.vectorcore.pitt.edu/upgene.htm	[108]

## 5 Summary

Significant advances have been made in the past decade toward revealing the role of codon bias and synonymous codon substitution and the impact on regulating native gene expression, mRNA secondary structure, and protein function and structure. Codon usage bias generally reflects a balance between mutational forces and natural selection, leading to optimal translational efficiency. Limiting tRNA isoacceptor pools during translation can have a significant, negative effect on the accuracy of translation by impacting the progression of ribosomes on mRNA, leading to ribosomal stalling or queuing, premature translational termination, translational frame-shifting, and amino acid mis-incorporation. Clearly, the extensive body of information included in genomic and proteomic databases has allowed for comprehensive surveys of genes and their proteins, and has redefined the roadmap that is used for efficient protein translation. Well-adapted codons, that is, preferred codons, could confer a metabolic advantage by selecting for translation efficiency and reducing the impact of misfolded proteins. Thus, with a view toward developing optimal strategies for synthetic gene design, increasing the relative AU codon content (i.e., lowering mRNA hairpin structure stability) in the termini of an ORF (particularly the 5′-end) can lead to dramatic improvements in expression levels [99]. The roadblocks to heterologous expression can be alleviated by considering sequence GC content, and thus, codon bias relative to the host expression system and the native gene sequence. Improvements can be augmented by avoiding strong mRNA secondary structures primarily at the 5′-end of coding sequences, allowing for more stable translation initiation complexes and ensuring impediment-free launches of ribosomes on mRNA required for efficient peptide translation and elongation.

## Abbreviations

AU: adenine:thymidine
CAI: codon adaptation index
GC: guanidine:cytosine
GFP: green fluorescent protein
ORF: open reading frame
RBS: ribosomal binding site
SD: Shine and Dalgarno
